# Neuroendocrine prostate cancer has distinctive, non-prostatic HOX code that is represented by the loss of HOXB13 expression

**DOI:** 10.1038/s41598-021-82472-1

**Published:** 2021-02-02

**Authors:** Siyuan Cheng, Shu Yang, Yingli Shi, Runhua Shi, Yunshin Yeh, Xiuping Yu

**Affiliations:** 1 Department of Biochemistry and Molecular Biology, LSU Health-Shreveport, Shreveport, LA USA; 2 Department of Medicine, LSU Health-Shreveport, Shreveport, LA USA; 3 Pathology and Laboratory Medicine Service, Overton Brooks VA Medical Center, Shreveport, LA USA; 4 Department of Urology, LSU Health-Shreveport, Shreveport, LA USA

**Keywords:** Prostate cancer, Gene expression, Data mining

## Abstract

HOX gene-encoded homeobox proteins control body patterning during embryonic development; the specific expression pattern of HOX genes may correspond to tissue identity. In this study, using RNAseq data of 1019 human cancer cell lines that originated from 24 different anatomic sites, we established HOX codes for various types of tissues. We applied these HOX codes to the transcriptomic profiles of prostate cancer (PCa) samples and found that the majority of prostate adenocarcinoma (AdPCa) samples sustained a prostate-specific HOX code whereas the majority of neuroendocrine prostate cancer (NEPCa) samples did not, which reflects the anaplastic nature of NEPCa. Also, our analysis showed that the NEPCa samples did not correlate well with the HOX codes of any other tissue types, indicating that NEPCa tumors lose their prostate identities but do not gain new tissue identities. Additionally, using immunohistochemical staining, we evaluated the prostatic expression of HOXB13, the most prominently changed HOX gene in NEPCa. We found that HOXB13 was expressed in both benign prostatic tissues and AdPCa but its expression was reduced or lost in NEPCa. Furthermore, we treated PCa cells with all trans retinoic acid (ATRA) and found that the reduced HOXB13 expression can be reverted. This suggests that ATRA is a potential therapeutic agent for the treatment of NEPCa tumors by reversing them to a more treatable AdPCa.

## Introduction

Homeobox proteins (HOXs) are transcription factors that regulate anterior–posterior patterning during embryogenesis^1^. In humans, there are 39 HOX genes organized into 4 clusters. Each cluster is composed of paralogous genes 1–13^1^. The 3′–5′ organization of these genes reflects their temporal and spatial expression pattern during embryonic morphogenesis. The 3′ HOX genes are activated first in anterior embryonic domains followed by the expression of 5′ genes in caudal areas^1,2^. The expression of certain HOX genes in a given anatomic region determines its tissue specificity; as such, that region’s combined expression pattern of HOX genes is called its “HOX code”^3^.

Prostate cancer (PCa) is the most diagnosed cancer among American men. In 2020, 191,930 men in the US are predicted to be diagnosed with PCa and 33,330 to die from this disease^4^. Neuroendocrine prostate cancer (NEPCa) is an aggressive type of PCa. After androgen deprivation therapy fails, about 30% of PCa patients acquire the neuroendocrine (NE) phenotype^5^. Although the cell of origin of NEPCa has not been conclusively determined, accumulating evidence indicates that NEPCa cells arise from the trans-differentiation of prostate adenocarcinoma, the probable first step of NEPCa development^6^. Currently, there is no effective treatment for PCa with a prominent NE phenotype. Thus, understanding how PCa progresses into NEPCa could have important diagnostic and treatment implications.

HOXB13, the most posterior HOXB gene, is expressed in the caudal region of developing embryos, including the tailbud, parts of spine, and hindgut as well as the urogenital sinus, from which the prostate is developed^7,8^. In adult prostates, especially in luminal epithelial cells, HOXB13 is highly expressed^9–11^. Consistent with the general role of HOX proteins in cell fate determination and tissue differentiation^1^, HOXB13 regulates prostate development and epithelial cell differentiation in normal tissues^9,12^. HOXB13 is also involved in prostate carcinogenesis and cancer progression^13^. Studies have shown that mutations in the HOXB13 gene are associated with PCa^13^. Additionally, microarray-based transcriptome analyses revealed the progressive upregulation of HOXB13 in PCa cells, and HOXB13 is considered a marker for prostatic cancer origins, to distinguish metastatic PCa from other cancers^14,15^. However, it remains unclear whether HOXB13 plays a tumor suppressive or pro-oncogenic role in PCa. On the one hand, HOXB13 expression is elevated in castrate-resistant PCa and the induced expression of HOXB13 promotes androgen-independent growth of LNCaP cells^16^. On the other hand, constitutive expression of HOXB13 suppresses colony formation in these cells^17^.

In this study, we established a list of HOX codes for various tissue sites and applied them to PCa samples. We found that NEPCa samples had different HOX codes from AdPCa samples. Also, using immunohistochemical staining, we evaluated the protein expression of HOXB13, the most prominently changed HOX gene in NEPCa. The results revealed that HOXB13 expression was maintained in AdPCa but was reduced or lost in NEPCa. Finally, we report that the decreased HOXB13 expression in PCa cells can be reverted.

## Results

### Establishment of HOX codes of various tissue origins

**Figure 1:**
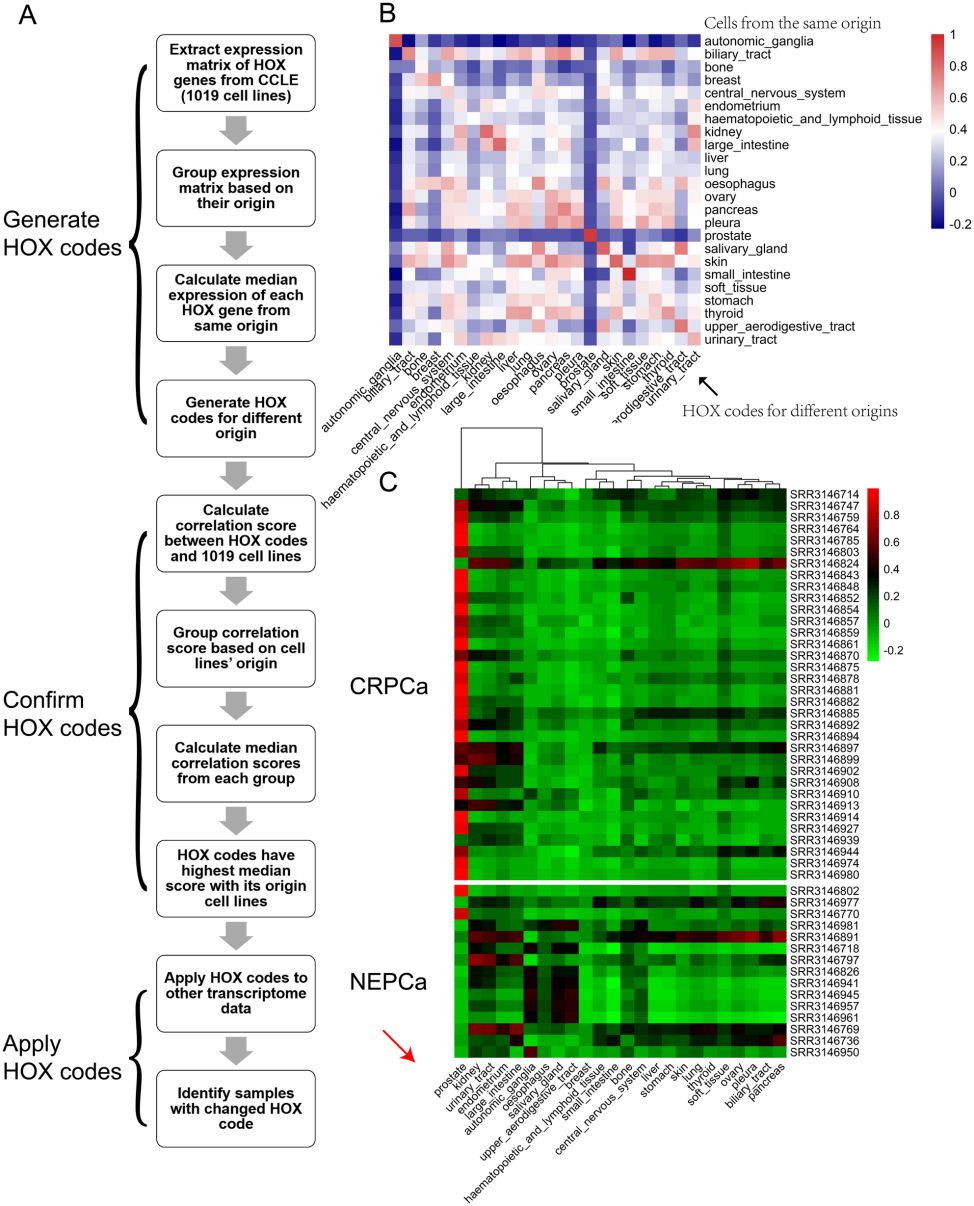
The loss of prostate HOX code in NEPCa tumors. (**A**) The workflow of establishing the HOX codes of different tissues. (**B**) The correlation of the cell lines’ tissue origins (X-axis) and their HOX codes (Y-axis). Majority tissues correlated well with their HOX codes. It is noteworthy that prostate tissue displayed a high correlation with its HOX code (**C**) AdPCa and NEPCa tumors displayed high and low correlation with prostate HOX code, respectively.

It has been suggested that different tissues have unique HOX codes. Using the RNA-seq data collected by the Cancer Cell Line Encyclopedia (CCLE)^18^, we analyzed the expression of HOX genes in 1019 cell lines that were derived from various tissue origins and calculated the HOX code for each tissue type. The workflow is presented in Fig. 1A and the median correlation scores between cell lines of the same tissue origin and the HOX codes of various tissues are presented as a heatmap (Fig. 1B). This heatmap reflects how closely each tissue type is correlated with its HOX code. As shown in Fig. 1B, most tissue types had a rather strong correlation with their calculated HOX codes. Among these tissues, the most notable was the prostate group (consisting of 8 cell lines), followed by autonomic ganglia and skin, all displaying a strong correlation with their HOX code. To further validate the HOX codes, we applied the CCLE-derived HOX codes to TCGA datasets of various cancer types and found that most HOX codes displayed high correlation with the tissues they represented (sFigure 1). Among these, prostatic HOX code is a prominent and exclusive one.

### NEPCa tumors display a change in HOX code

The strong correlation between the tissue types and their HOX codes suggests that the HOX codes could be used to identify the tissue origin of a given sample. Additionally, HOX codes could be used to study how closely a sample is related to its tissue origin. A “lineage-keep” sample would display a high correlation with the HOX code of its tissue origin, whereas a “lineage-switch” sample would show a low correlation. Therefore, we applied the HOX codes to a RNA-seq dataset of human PCa including 34 CRPCa and 15 NEPCa samples^5^. The correlation scores between the expression of HOX genes in each sample (row) and the HOX codes for various tissues (column) were visualized in a heatmap (Fig. 1C). The vast majority (31/34) of CRPCa samples displayed a high correlation (Pearson correlation score > 0.3) with the HOX code of prostate but not of other tissues. More importantly, the majority of (13/15) NEPCa samples showed a low or negative correlation with the prostate HOX code. Similar results were also observed in other NEPCa datasets including SU2C dataset^19^ (sFigure 2A), GSE32967^20^ (sFigure 2B), GSE41192^21^ (sFigure 2C), GSE66187^22^ (sFigure 2D). While NEPCa tumors displayed a loss of the prostate specific HOX code, they did not show a consistent correlation with the HOX codes of any other tissues (Fig. 1 and sFigure 2).

Taken together, these results indicate that HOX code, to a certain extent, could reflect the identity of tissues and that the loss of the prostate specific HOX code in NEPCa reflects a loss of prostatic identity in these tumors.

### The expression of HOXB13 is decreased in NEPCa

Among all the HOX genes, HOXB13 displayed the highest mRNA level in prostate specimens (sFigure 3A). Compared with other posterior HOX genes (HOX13s), HOXB13 also displayed a more consistent expression pattern in TCGA prostatic samples (sFigure 3B). Therefore, we specifically examined HOXB13 expression in prostatic tissues.

**Figure 2:**
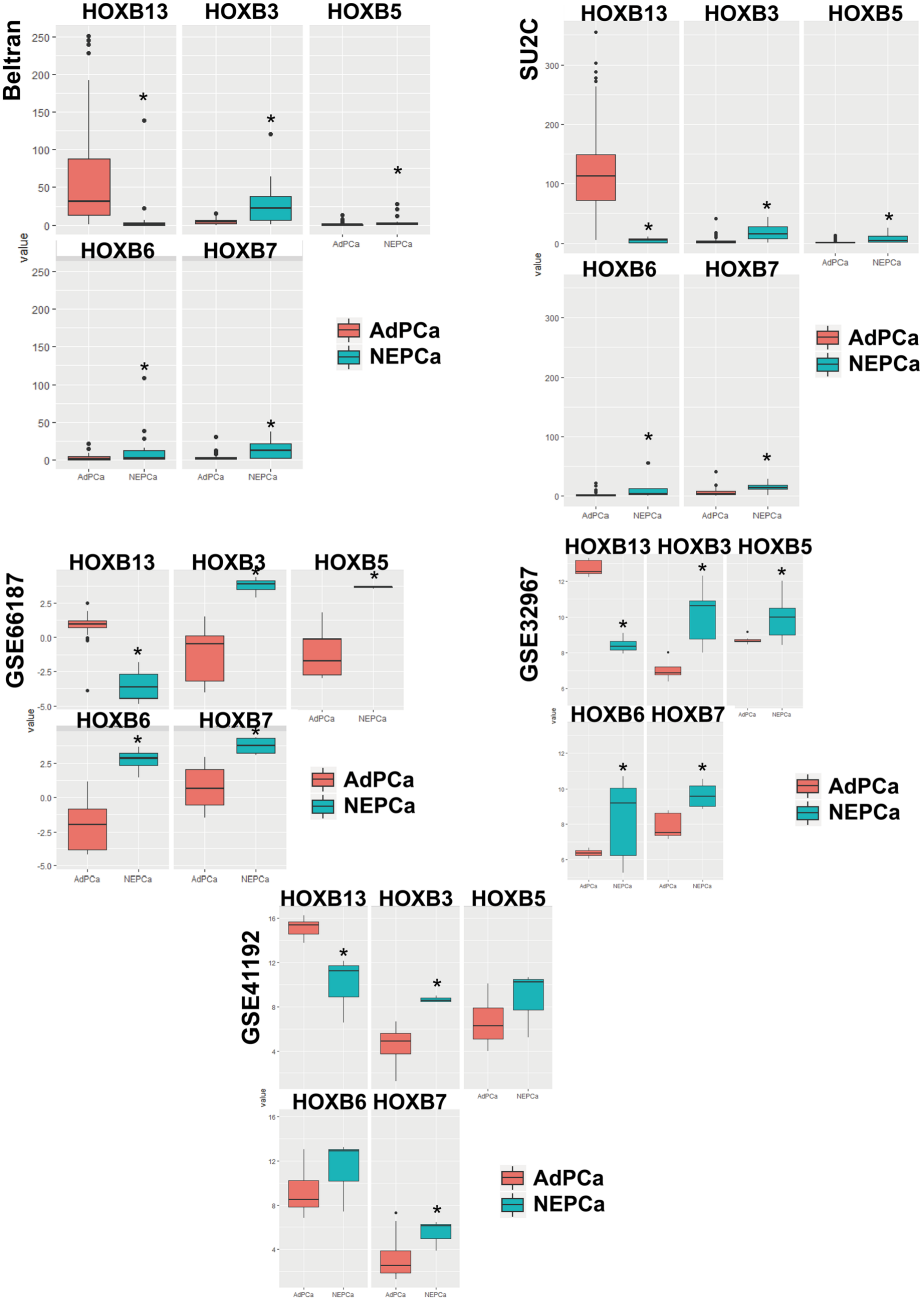
The expression of HOXB genes in AdPCa and NEPCa samples. The expression data of HOXB13, B3, B5, B6, and B7 were extracted from NEPCa Beltran dataset^5^, SU2C^19^, GSE66187^22^, GSE32967^20^ and GSE41192^21^. HOXB13 level was lower in NEPCa than AdPCa samples whereas the levels of HOXB3, B5, B6, and B7 were higher in NEPCa samples. *p < 0.05, t-test.

Analysis of PCa TCGA dataset indicates that consistent with a previous report^23^, the mRNA level of HOXB13 is higher in PCa than in normal prostatic tissues (sFigure 3C). Additionally, analysis of NEPCa RNA-seq datasets^5,19–22,24^ indicates that HOXB13 is the most changed HOX gene in NEPCa when compared with AdPCa. As shown in Fig. 2, the mRNA level of HOXB13 was lower in NEPCa samples than AdPCa in all the NEPCa datasets examined. In contrast, the mRNA levels of several other members of the HOXB family including HOXB3, HOXB5, HOXB6 and HOXB7 were higher in NEPCa than AdPCa.

**Figure 3:**
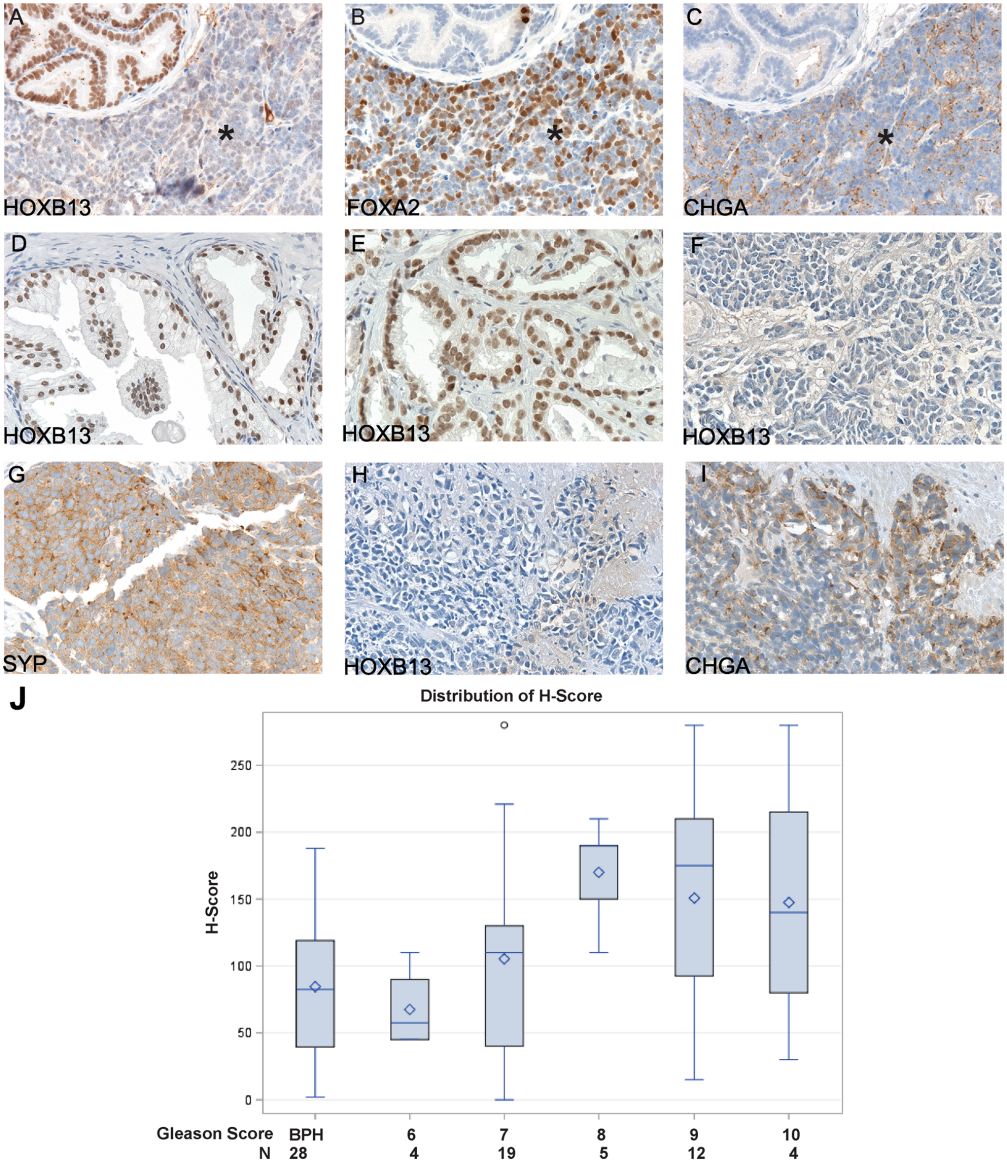
Immunohistochemical staining to evaluate the expression of HOXB13 in prostatic tissues. (**A**–**C**) Serial sections of a TRAMP NEPCa tumor displayed low HOXB13 expression in NEPCa area (indicated by * sign) in contrast to the positive staining in focal PIN. NEPCa markers Chromogranin A (CHGA) and FOXA2 were stained positive in NEPCa cells but negative in PIN. (**D**) HOXB13 expression in BPH, n = 28. (**E**) HOXB13 expression in AdPCa, n = 44. (**F**–**I**) Human NEPCa tumors demonstrated reduced (n = 1) or negative (n = 8) staining of HOXB13. These NEPCa tumors were stained positive for Synaptophysin (SYP) or Chromogranin A (CHGA). (**J**) Quantification of HOXB13 immunohistochemical staining with H-scores in benign prostatic hyperplasia (BPH) and prostatic acinar adenocarcinomas with a Gleason score of 6 (3 + 3), 7 (3 + 4 and 4 + 3), 8 (4 + 4), 9 (4 + 5 and 5 + 4), and 10 (5 + 5). The H score of HOXB13 immunohistochemical staining was correlated with Gleason score, Spearman correlation test, Rho = 0.348, p < 0.05.

Further, immunohistochemical (IHC) staining was conducted to assess the protein expression of Hoxb13. As shown in Fig. 3A–C, in murine PCa derived from TRAMP mice (n = 10), Hoxb13 expression was detected in prostatic intraepithelial neoplasia (PIN) lesions (Fig. 3A). However, the expression of Hoxb13 was decreased in the NEPCa area (Fig. 3A), which was highlighted by the positive stain of NEPCa markers Foxa2 (Fig. 3B) and chromogranin A (Fig. 3C).

The expression of HOXB13 was also evaluated in human prostatic tissues including benign prostate, AdPCa and NEPCa. As shown in Fig. 3D–I, HOXB13 expression was detected in both benign prostatic tissues (Fig. 3D) and prostate adenocarcinomas (AdPCa, Fig. 3E). In benign prostate samples, HOXB13 was stained positive in 26 of 28 BPH. Compared to that of BPH, the intensity of HOXB13 staining was greater in adenocarcinomas with Gleason scores of 7, 8, 9, and 10 albeit not statistically significant, possibly due to the wide variations in H-score observed in both BPH and the adenocarcinomas (Fig. 3J). The largest variation in H-score, ranged from 15 to 280, was noted in adenocarcinoma with Gleason score of 9. The highest H-score of 280 was found in adenocarcinomas with Gleason scores of 9 and 10. There was a positive correlation between the expression of HOXB13 (H-score) and Gleason scores of AdPCa (Spearman correlation test, Rho = 0.348, p < 0.05).

In contrast to the positive stain in AdPCa, no HOXB13 immunohistochemical reactivity was observed in 8 of 9 NEPCa including 8 small cell carcinoma and 1 neuroendocrine carcinoma, not otherwise specified. Of the 9 NEPCa, there was only 1 small cell carcinoma stained weakly and focally (H-score of 2) with HOXB13 immunomarker.

In summary, HOXB13 expression was detected in both benign prostatic tissues and prostate adenocarcinomas but its expression was lost or reduced in NEPCa tumors.

### HOXB13 is expressed in prostatic luminal epithelial cells

**Figure 4:**
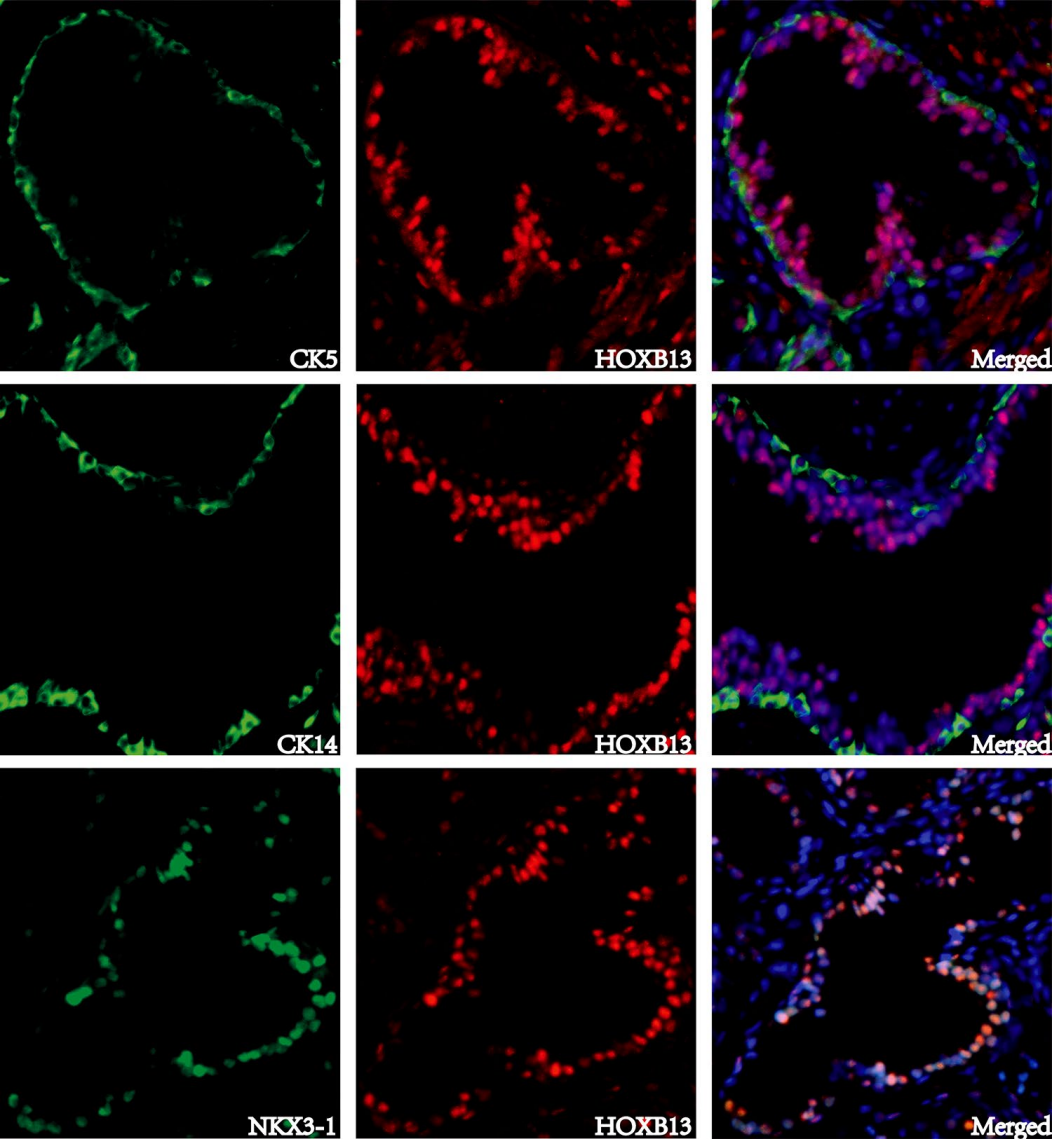
Dual immunofluorescence staining of HOXB13 with prostate basal epithelial markers cytokeratin 5 (CK5), cytokeratin 14 (CK14), or with prostate luminal epithelial markers NKX3-1. HOXB13 expression was detected in prostate luminal but not basal epithelial cells.

Dual immunofluorescence staining was conducted to examine the expression pattern of HOXB13 in prostatic glands. As shown in Fig. 4, the expressions of HOXB13 and basal epithelial cell marker (Cytokeratin 5 or Cytokeratin 14) were mutually exclusive. However, HOXB13 was co-expressed with prostate luminal epithelial cell marker NKX3-1. The luminal expression of HOXB13 was further supported by an analysis of a single cell RNA-seq dataset of normal human prostate (GSE120716), where HOXB13 positive cells were enriched in luminal epithelial subpopulation (sFigures 4A,B)^25,26^. A similar result was seen in a single cell RNA-seq analysis of mouse prostate (sFigure 4C)^27^.

### DU145, a commonly used PCa cell line, is more similar to NEPCa cell line H660

**Figure 5:**
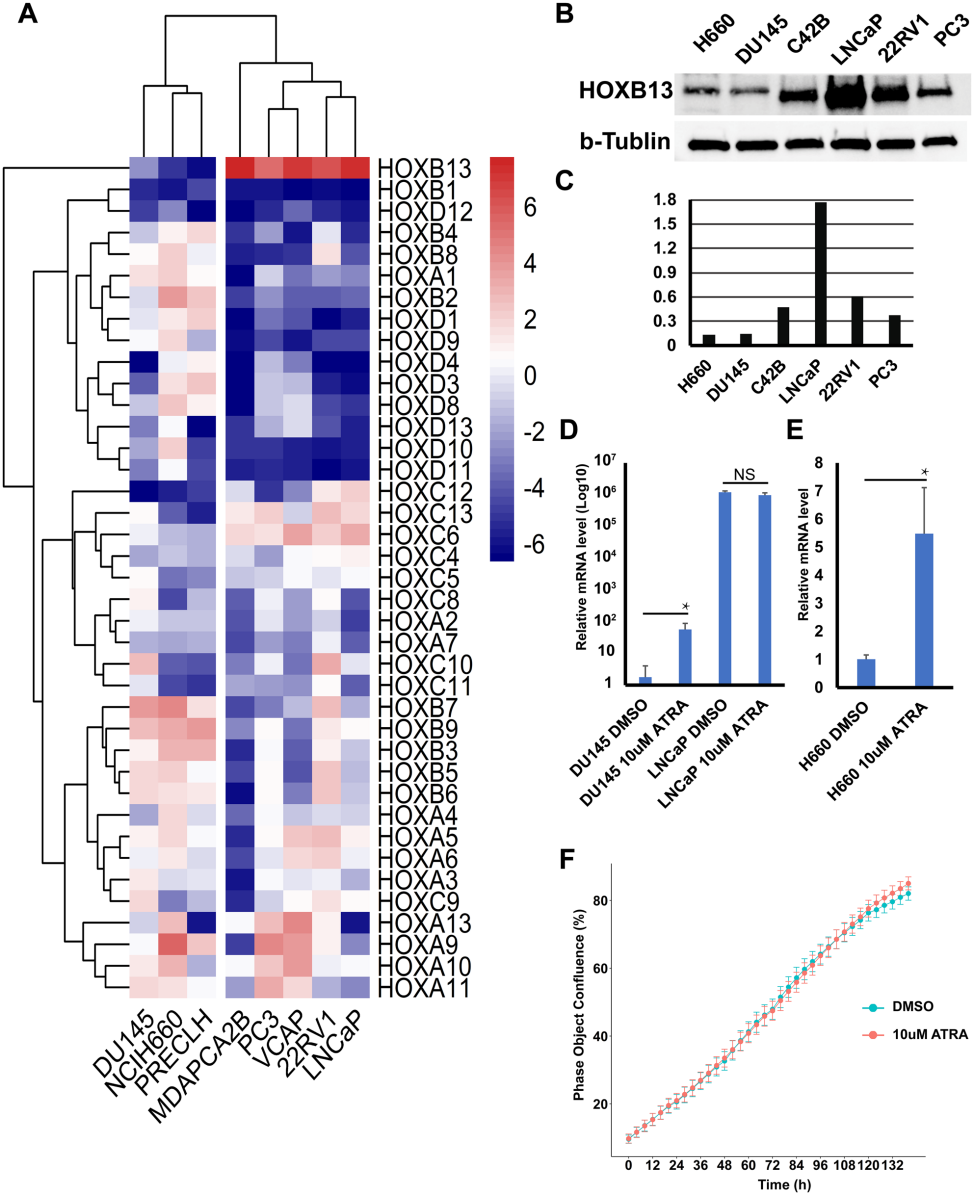
The decreased HOXB13 expression in PCa cells can be reverted. (**A**) A heatmap of HOX genes in PCa cell lines. DU145, H660, and PRECLH cell lines were clustered together, away from the other PCa cell lines. HOXB13 levels were lower in these three cell lines. (**B**) Western blot analysis confirmed the low HOXB13 protein levels in H660 and DU145 cells. (**C**) The quantification of Western blot result. (**D** and **E**) RT-qPCR to assess the mRNA levels of HOXB13 in DU145, LNCaP and H660 cells that were treated with all-trans retinoic acid (ATRA, 10^–5^ M). ATRA induced the mRNA expression of HOXB13 in DU145 and H660 but not in LNCaP cells. (**F**) IncuCyte live-cell imaging analysis indicates that 10 μM ATRA did not affect the proliferation of DU145 cells (p > 0.05 in all time points, t-test).

HOX code analysis of the eight prostatic cell lines that are collected in the CCLE dataset^18^ indicated that H660 (a NEPCa cell line), DU145 (a widely used AdPCa cell line), and PRECLH cell lines displayed a low association with the prostate HOX code whereas the other five PCa cell lines displayed a strong correlation with prostate HOX code (sFigure 5). This is consistent with the results of clustering analysis of HOX genes in these cell lines, which revealed that the cell lines that have lost prostate HOX code (DU145, H660, and PRECLH) were clustered together, distinct from the other five cell lines (Fig. 5A). Compared with that of the other five cell lines, the mRNA levels of HOXB13 were lower in the three cell lines that have lost prostate HOX code. The reduced HOXB13 expression in H660 and DU145 cells was confirmed by Western Blot analysis (Fig. 5B and C). These results indicate that DU145, labeled as an AdPCa cell line, is more similar to a NECPa cell line than other AdPCa cell lines with regard to its HOX code.

### The reduced expression of HOXB13 in PCa cells can be reverted

Accumulating evidence supports that NEPCa tumors emerge via the trans-differentiation of AdPCa and that transcriptome reprogramming plays a key role in this process. Our HOX code analysis suggests the trans-differentiation is accompanied with a change in HOX code. Here using HOXB13, the most prominently changed HOX gene, as an indicator of epigenetic reprogramming in NEPCa, we tested whether the reduced HOXB13 expression in NEPCa cells could be reverted. We treated DU145 and NCI-H660 cells with all-trans retinoic acid (ATRA), a commonly used agent to induce the differentiation of stem cells^28^. The established NEPCa NCI-H660 cell line was used in this experiment. Because the DU145 cell line was shown to display a similar HOX code to H660 and previous studies have shown that DU145 cells have some NEPCa features^29^, this cell line was also included in the ATRA experiments. The AdPCa LNCaP cells, which express high level of HOXB13, were used as a control cell line. As shown in Fig. 5D and E, the mRNA levels of HOXB13 were induced in both H660 and DU145 but not LNCaP cells that were treated with ATRA. However, ATRA did not induce the protein expression of HOXB13 in these cells (data not shown). To further evaluate whether ATRA has any functional roles in altering the proliferation of PCa cells, we conducted cell proliferation assays and found that ATRA at 10 μM did not significantly change the proliferation of DU145 cells (Fig. 5F) or LNCaP cells (data not shown). Taken together, these data indicate that ATRA can revert the mRNA expression of HOXB13 in NEPCa but not AdPCa cells. However, ATRA could not induce the protein levels of HOXB13 nor affect the proliferation of PCa cells.

## Discussion

In this paper, we report that HOXB13 is expressed in benign prostatic tissues and prostate adenocarcinomas, but its expression is decreased or lost in both human and mouse NEPCa. These data indicate that the loss of HOXB13 expression is a common feature of NEPCa.

During embryo development, a nested and partially overlapping HOX expression pattern is established. A change of HOX expression results in deficiencies in axial formation and the loss of tissue identity^1^. In this study, using transcriptomic data of 1019 cell lines, we established a method to calculate the HOX codes for 24 tissues and applied the HOX codes to PCa RNA-seq datasets. Using this method, we identified samples that uphold or lose prostate identity. We showed that prostate HOX code was sustained in the majority of AdPCa samples. This result is consistent with previous reports that HOXB13 is a PCa (adenocarcinoma) marker^14^. In contrast, the majority of NEPCa tumors and the NEPCa cell line NCI-H660 displayed low correlation with the HOX code of prostates, indicating a loss of prostate identity in NEPCa cells. DU145, an AdPCa cell line that has certain NEPCa features^29^, also displayed a loss of HOX code. Taken together, these data suggest that during NEPCa development, prostate identity is gradually lost in PCa cells.

Though NEPCa tumors seemed to lose prostate identity, they did not show consistent correlation with the HOX codes of any other tissues, suggesting that they have not acquired a clear-cut new identity. This is in line with the consensus that NEPCa cells lose prostate differentiation, but this notion is not in complete agreement with the proposed acquisition of neuronal features in NEPCa. Our observation of the lack of clear-cut identity of NEPCa adds to this line of knowledge. Although the acquired neuronal “features” in some NEPCa tumors resemble neuronal characteristics in certain aspects, NEPCa cells have not acquired the neuron “identity”.

Additionally, our HOX code method provides a unique technique for studying lineage switching during cancer progression. By applying the HOX codes to the transcriptome data of various prostate cell lines, we identified three cell lines that lose the prostate-specific HOX code. Among these, H660 is a NEPCa cell line and DU145 is a commonly used androgen-independent AdPCa cell line. Analysis of the HOX expression profile indicates that DU145 is more similar to NEPCa (H660) than other AdPCa cell lines. Given the NEPCa features in DU145 cells, these cells may represent a unique state of PCa—they have started losing prostatic differentiation but have yet to gain a full NE phenotype. This cell line could be a good model system for inducing NE differentiation.

Currently, there are no effective treatment options against NEPCa. A possible therapeutic approach is to revert the lineage switch of NEPCa. By inducing prostatic differentiation in NEPCa cells, we might be able to convert them back to the AdPCa state. For this purpose, we treated HOXB13-low NEPCa (NCI-H660) and NEPCa-like (DU145) cells with retinoic acid, a commonly used agent to induce differentiation^30^. We found that retinoic acid induced the mRNA expression of HOXB13 in both H660 and DU145 cell lines, but not in AdPCa LNCaP cells. This indicates that the loss of HOXB13 expression during the development of NEPCa is reversible. This notion also supports the trans-differentiation theory of NEPCa. However, ATRA did not induce the protein expression of HOXB13 in DU145 cells, suggesting the involvement of post-transcriptional regulatory mechanisms in HOXB13 expression. This may also explain the discrepancy between the mRNA and protein levels of HOXB13 in PC3 cells.

Our observation of the effects of ATRA is consistent with previous reports that ATRA induces the expression of HOXB13 in DU145 cells and inhibits NEPCa in TRAMP mice, PCa mouse models^31,32^. We showed here that ATRA reverts the lost expression of HOXB13 in NEPCa cells, providing evidence supporting the possibility of inducing prostate differentiation in NEPCa cells. Further research is warranted to study the therapeutic effects of retinoic acids and other differentiation-inducing agents in the treatment of NEPCa.

## Materials and methods

### Establishment and validation of HOX codes of different tissue origins

To establish the HOX code for each tissue origin, we extracted the expression matrix (FKPM) of 39 HOX genes from CCLE dataset. This dataset contains RNA-seq data of 1019 cell lines. These cell lines were grouped based on their tissue origin information. The median expression of each HOX gene in the grouped samples was calculated to generate a HOX code for each tissue origin. We then applied the HOX codes (Supplementary Table 1) to each of the 1019 cell lines by calculating the Pearson correlation scores between the expression of HOX genes in that cell line and the HOX codes of various tissue origins. A heatmap was constructed to visualize the median correlation score in each group of samples (X-axis) and their correlation with the HOX codes of various tissue origins (Y-axis). To validate the HOX codes, we applied the HOX codes to TCGA datasets of various types of cancer. We also applied the HOX codes to publicly available NEPCa datasets and calculated the Pearson correlation scores to reflect the correlation between the HOX expressions in each prostatic sample and the HOX codes of various tissue origins.

### Sample collection

De-identified human prostate tissue specimens were obtained from LSU Health-Shreveport Biorepository Core, Overton Brooks VA Medical Center, Ochsner Health System Biorepository and Tissue for Research as described previously^33^. The specimens used in this research include 28 benign prostate hyperplasia, 44 AdPCa, and 9 NEPCa tissues. All the tissues were used in accordance with LSU Health-Shreveport IRB protocols. Archived tissue sections of TRAMP tumors were used for this study.

### Immunohistochemical and immunofluorescence staining

Immunostaining was performed using Vectastain elite ABC peroxidase kit (Vector Laboratories, Burlingame, CA) as described previously^33^. Primary antibodies include HOXB13 and Chromogranin A (CHGA) (sc-28333 and sc-1488 respectively, Santa Cruz Biotechnology, Dallas, TX), FOXA2 (ab 23306-100, Abcam, Cambridge, MA), cytokeratin 5 (CK5) and 14 (CK14) (904,801 and 905,501 respectively, BioLegend, San Diego, CA). The tissue sections were counterstained, mounted, and imaged with a Zeiss microscope (White Plains, NY). The staining was evaluated using a semiquantitative H-score. Immunofluorescence staining was imaged with a Nikon fluorescence microscope (Melville, NY).

### Bioinformatics analyses

The mRNA expression data of HOXB genes were extracted from publicly available NEPCa datasets including RNA-seq datasets (from Beltran’s study^5^, SU2C^19^, and CCLE^18^) and microarray datasets (GSE66187^22^, GSE32967^20^, and GSE41192^21^). The Beltran dataset contains 49 PCa samples including 34 AdPCa and 15 NEPCa^5^. The SU2C dataset contains 113 AdPCa and 5 NEPCa samples^19^. GSE66187 contains 4 NEPCa and 20 AdPCa samples^22^. GSE32967 contains 14 NEPCa and 8 AdPCa samples^20^. GSE41192 contains 3 NEPCa and 29 AdPCa samples^21^. The expression of HOXB13 was compared between AdPCa and NEPCa samples in these datasets. Data manning was performed by using RStudio. Heatmaps were generated by using “Pheatmap” package and boxplots using “ggplot2” package.

### Cell culture and drug treatment

LNCaP, DU145 and H660 cells were obtained from ATCC and cultured in RPMI1640 supplemented with 10% FBS and Prostalife media (LifeLine Cell Technology, Oceanside, CA), respectively. All trans retinoic acid (Sigma Aldrich, St. Louis, MO) was dissolved in DMSO and diluted (1:2000) with cell culture media. The cells were treated with retinoic acid at a final concentration of 10^–5^ M for 1–4 days.

### Cell proliferation assay

IncuCyte S3 live cell imaging system (Essen BioScience, Ann Arbor, MI) was used to evaluate the effects of ATRA on the cell proliferation. PCa cells (1000 cells/well) were seeded in 96-well plate and cultured in media containing DMSO or 10 μM ATRA. Cell culture media were refreshed every two days. Images were taken every four hours and the cell proliferation was monitored based on the cell confluency.

### RNA extraction and RT-qPCR

RNA was extracted by using RNeasy Mini Kit (Germantown, MD). Reverse transcription and qPCR were conducted by using iScript Reverse Transcription Supermix and SYBR green PCR Supermix (BioRad, Hercules, CA). GAPDH was used to normalize the gene expression. Primer sequences were listed in sTable 2.

### Western blot

Cells were collected in PBS and lysed in passive lysis buffer (Promega, Madison, WI). Equal amounts of protein were loaded for Western blot analyses. ProSignal Dura ECL Reagent (Genesee Scientific, San Diego, CA) and Chemidoc (Bio-Rad, Hercules, CA) were used to visualize the proteins. Beta-tubulin was used as loading control.

### Statistical analyses

T-test was used to compare the gene expression in AdPCa and NEPCa samples. Spearman Correlation Coefficient test was used to analyze the correlation between tumor grade and protein expression (H-score). p < 0.05 was considered statistically significant.

## Supplementary information

Supplementary Information.

Supplementary Tables.

## References

[1] Gehring WJ, Hiromi Y (1986). Homeotic genes and the homeobox. Annu. Rev. Genet..

[2] Krumlauf R (1994). Hox genes in vertebrate development. Cell.

[3] Kessel M, Gruss P (1991). Homeotic transformations of murine vertebrae and concomitant alteration of Hox codes induced by retinoic acid. Cell.

[4] Siegel RL, Miller KD, Jemal A (2020). Cancer statistics. CA Cancer J. Clin..

[5] Beltran H (2014). Aggressive variants of castration-resistant prostate cancer. Clin. Cancer Res..

[6] Zou M (2017). Transdifferentiation as a mechanism of treatment resistance in a mouse model of castration-resistant prostate cancer. Cancer Discov..

[7] Hankey, W., Chen, Z. & Wang, Q. Shaping chromatin states in prostate cancer by pioneer transcription factors. *Cancer Res.* (2020). 10.1158/0008-5472.CAN-19-3447 PMC7299826 32094298

[8] Brechka H, Bhanvadia RR, VanOpstall C, Vander Griend DJ (2017). HOXB13 mutations and binding partners in prostate development and cancer: function, clinical significance, and future directions. Genes Dis..

[9] Economides KD, Capecchi MR (2003). Hoxb13 is required for normal differentiation and secretory function of the ventral prostate. Development.

[10] Sreenath T, Orosz A, Fujita K, Bieberich CJ (1999). Androgen-independent expression of hoxb-13 in the mouse prostate. Prostate.

[11] VanOpstall C (2020). MEIS-mediated suppression of human prostate cancer growth and metastasis through HOXB13-dependent regulation of proteoglycans. Elife.

[12] Huang L, Pu Y, Hepps D, Danielpour D, Prins GS (2007). Posterior Hox gene expression and differential androgen regulation in the developing and adult rat prostate lobes. Endocrinology.

[13] Ewing CM (2012). Germline mutations in HOXB13 and prostate-cancer risk. N. Engl. J. Med..

[14] Edwards S (2005). Expression analysis onto microarrays of randomly selected cDNA clones highlights HOXB13 as a marker of human prostate cancer. Br. J. Cancer.

[15] Varinot J (2013). HOXB13 is a sensitive and specific marker of prostate cells, useful in distinguishing between carcinomas of prostatic and urothelial origin. Virchows Arch..

[16] Kim Y-R (2010). HOXB13 promotes androgen independent growth of LNCaP prostate cancer cells by the activation of E2F signaling. Mol. Cancer.

[17] Jung C, Kim R-S, Zhang H-J, Lee S-J, Jeng M-H (2004). HOXB13 induces growth suppression of prostate cancer cells as a repressor of hormone-activated androgen receptor signaling. Can. Res..

[18] Ghandi M (2019). Next-generation characterization of the cancer cell line encyclopedia. Nature.

[19] Robinson D (2015). Integrative clinical genomics of advanced prostate cancer. Cell.

[20] Tzelepi V (2012). Modeling a lethal prostate cancer variant with small-cell carcinoma features. Clin. Cancer Res..

[21] Clermont P-L (2015). Polycomb-mediated silencing in neuroendocrine prostate cancer. Clin. Epigenet..

[22] Zhang X (2015). SRRM4 expression and the loss of REST activity may promote the emergence of the neuroendocrine phenotype in castration-resistant prostate cancer. Clin. Cancer Res..

[23] Weiner, A. B. *et al.* Somatic HOXB13 Expression Correlates with Metastatic Progression in Men with Localized Prostate Cancer Following Radical Prostatectomy. *Eur. Urol. Oncol.* (2020). 10.1016/j.euo.2020.05.001 PMC7736205 32540218

[24] Cheng S, Yu X (2019). Bioinformatics analyses of publicly available NEPCa datasets. Am. J. Clin. Exp. Urol..

[25] Henry GH (2018). A cellular anatomy of the normal adult human prostate and prostatic urethra. Cell Rep..

[26] Kwon, O.-J. *et al.* De novo induction of lineage plasticity from human prostate luminal epithelial cells by activated AKT1 and c-Myc. *Oncogene*, 1–10 (2020). 10.1038/s41388-020-01487-6 PMC7704645 33009488

[27] Karthaus WR (2020). Regenerative potential of prostate luminal cells revealed by single-cell analysis. Science.

[28] Pagliuca FW (2014). Generation of functional human pancreatic β cells in vitro. Cell.

[29] Leiblich A (2007). Human prostate cancer cells express neuroendocrine cell markers PGP 9.5 and chromogranin A. Prostate.

[30] Summerbell D, Maden M (1990). Retinoic acid, a developmental signalling molecule. Trends Neurosci..

[31] Huss WJ, Lai L, Barrios RJ, Hirschi KK, Greenberg NM (2004). Retinoic acid slows progression and promotes apoptosis of spontaneous prostate cancer. Prostate.

[32] Liu Z (2012). ATRA inhibits the proliferation of DU145 prostate cancer cells through reducing the methylation level of HOXB13 gene. PLoS ONE.

[33] Cheng, S. *et al.* The expression of YAP1 is increased in high-grade prostatic adenocarcinoma but is reduced in neuroendocrine prostate cancer. *Prostate Cancer and Prostatic Diseases*, 1–9 (2020). 10.1038/s41391-020-0229-z PMC7572469 32313141

