# Supplementary material for: Neuroendocrine prostate cancer has distinctive, non-prostatic HOX code that is represented by the loss of HOXB13 expression
Source: Sci Rep. 2021 Feb 2;11:2778. doi: 10.1038/s41598-021-82472-1 (PMC7854582; doi:10.1038/s41598-021-82472-1)
Supplement: Supplementary file 1 — Supplementary Information. [file 41598_2021_82472_MOESM1_ESM.docx]

**Neuroendocrine prostate cancer has distinctive, non-prostatic HOX code that is represented by the loss of HOXB13 expression**

# Siyuan Cheng^1^, Shu Yang^1^, Yingli Shi^1^, Runhua Shi^2^, Yunshin Yeh^3,4^, Xiuping Yu^1,4^*

1. Department of Biochemistry & Molecular Biology, LSU Health-Shreveport, Shreveport, LA, USA
2. Department of Medicine, LSU Health-Shreveport, Shreveport, LA, USA
3. Pathology & Laboratory Medicine Service, Overton Brooks VA Medical Center, Shreveport, LA, USA
4. Department of Urology, LSU Health-Shreveport, Shreveport, LA, USA

*Correspondence to xyu@LSUHSC.edu

**
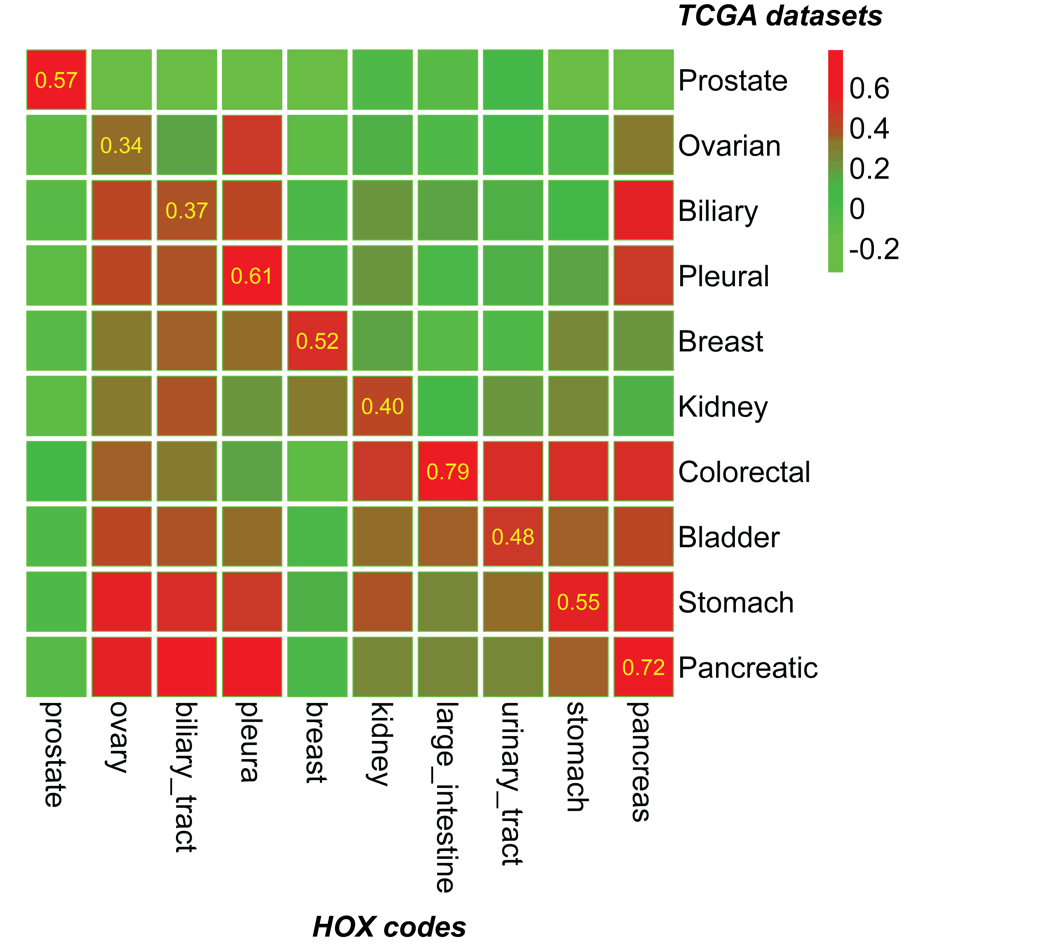
**

**sFigure 1** Validation of HOX codes. Heatmap was constructed by using the median correlation scores between TCGA cancer datasets (Rows) and HOX codes (columns). The HOX codes correlated well with their represented tissues.





**sFigure 2** Heatmaps to show the correlation (Pearson association) of PCa samples with the HOX codes of different tissue origins. Each row represents a PCa sample and each column represents the HOX code of a tissue origin. The columns of prostate HOX code are highlighted. The NEPCa samples are labeled with “*”.

**
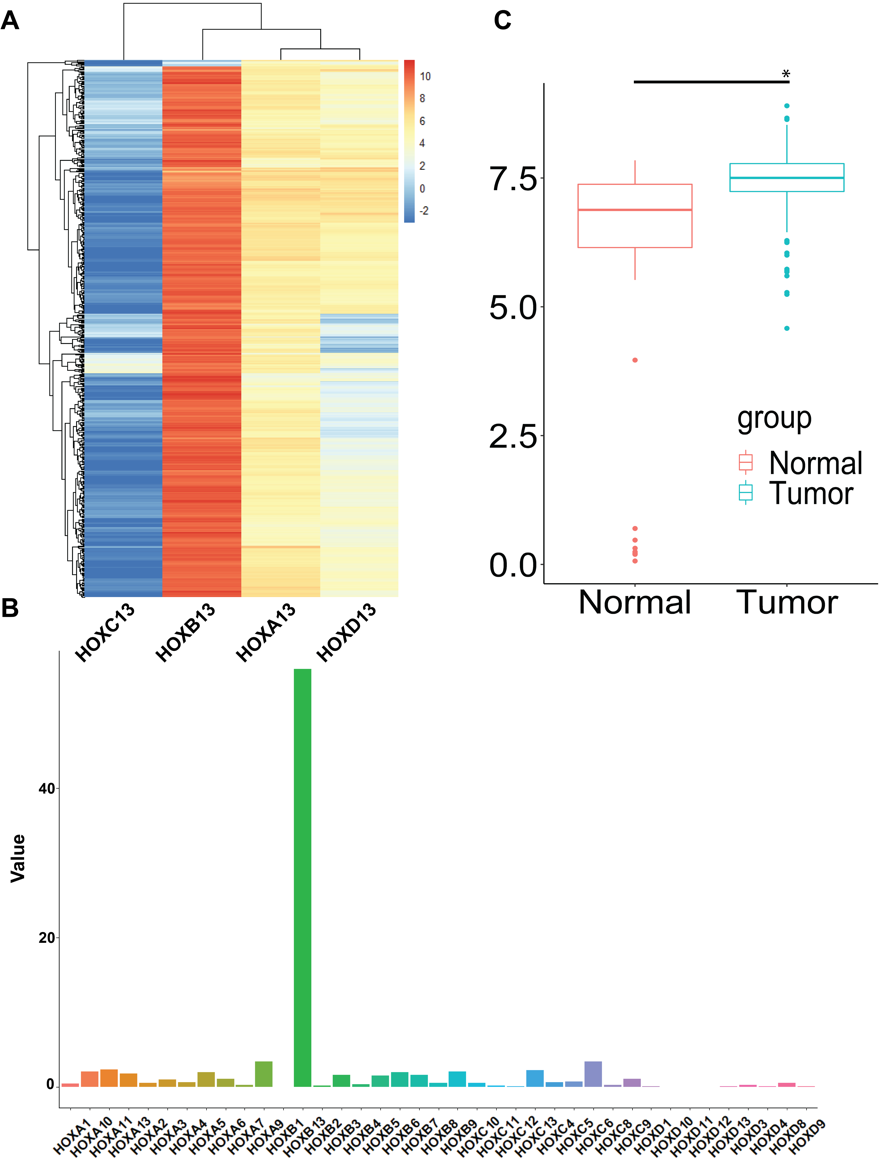
**

**sFigure 3** HOXB13 is the most prominently expressed HOX gene in prostate. (A) Heatmap of 4 posterior HOX genes (HOXA, B, C, D13) expression in TCGA prostate cancer dataset suggesetd HOXB13 has the highest expression among 4 genes. (B) Bar plot showing the value of prostatic HOX code. HOXB13 has the highest level among 39 HOX genes. (C) HOXB13 expression in normal prostate and PCa tissues (TCGA dataset), * p<0.05, t-test.

**
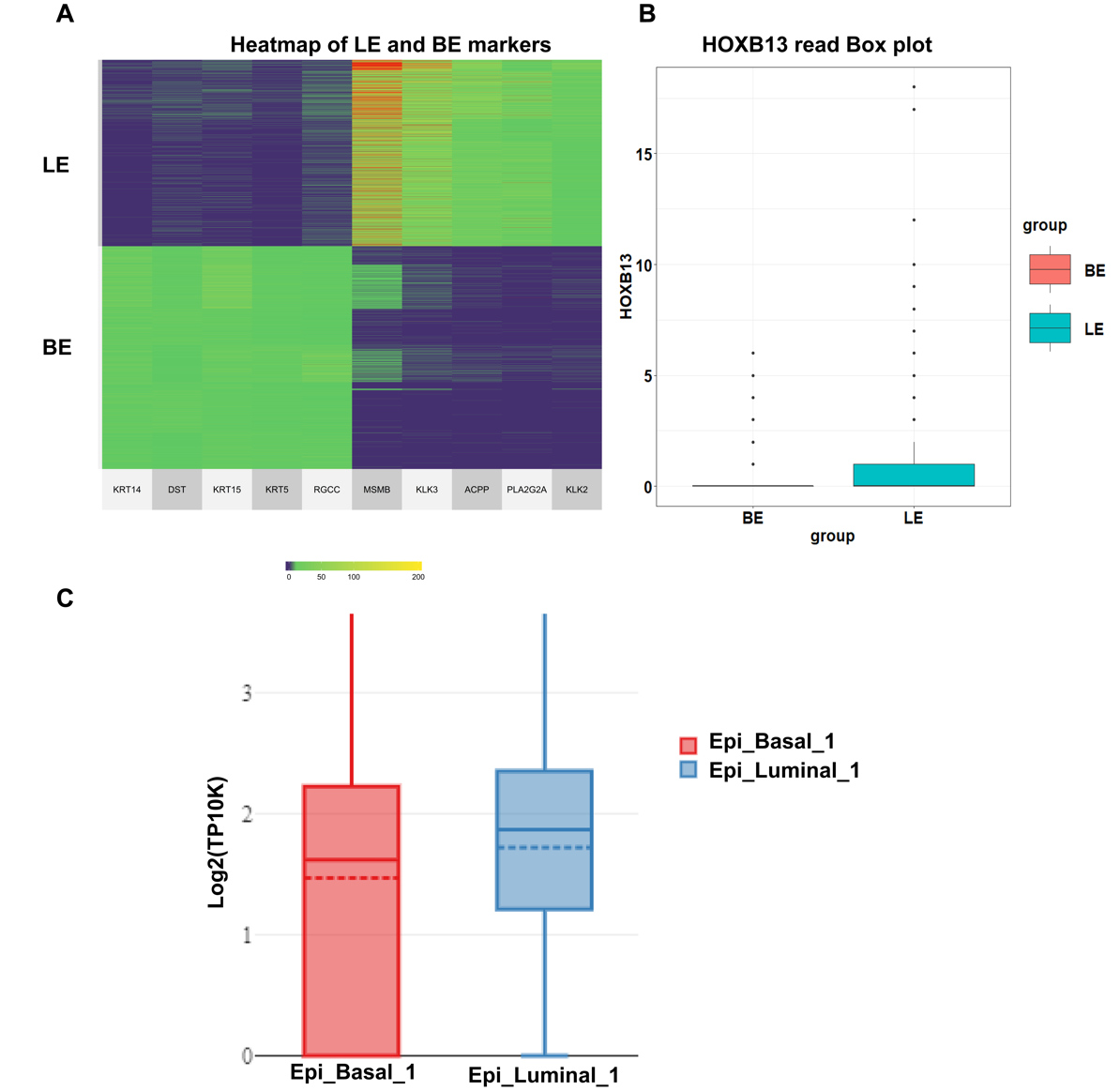
**

**sFigure 4** HOXB13 expression is enriched in luminal epithelial cells. Analysis of single cell RNA sequencing data (GSE120716^26^) indicated that HOXB13 was enriched in luminal epithelia (LE) compared to basal epithelia (BE), t-test, p<0.05. (A) Heatmap to show the different cell populations that were selected based on the expression of maker genes. (B) Boxplot to show the distribution of HOXB13 positive cells in luminal and basal cell populations. (C) Single cell RNAseq analysis of mouse prostates indicates that Hoxb13 positive cells are enriched in the luminal epithelia^28^.


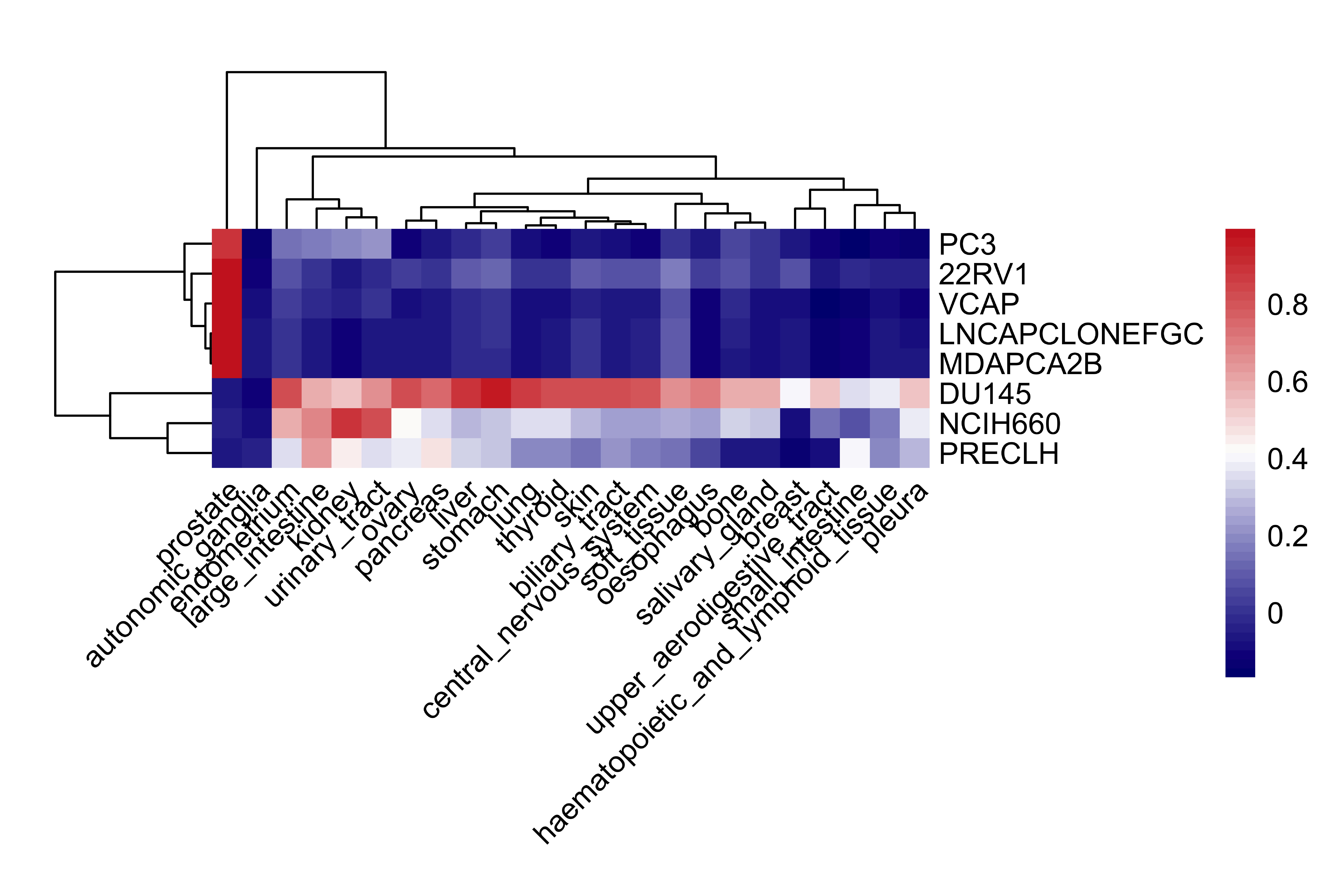


**sFigure 5** Heatmap to show the correlation (Pearson association) of PCa cell lines with the HOX codes of different tissue origins. NCI-H660 is a NEPCa cell line and the rest cell lines are AdPCa.


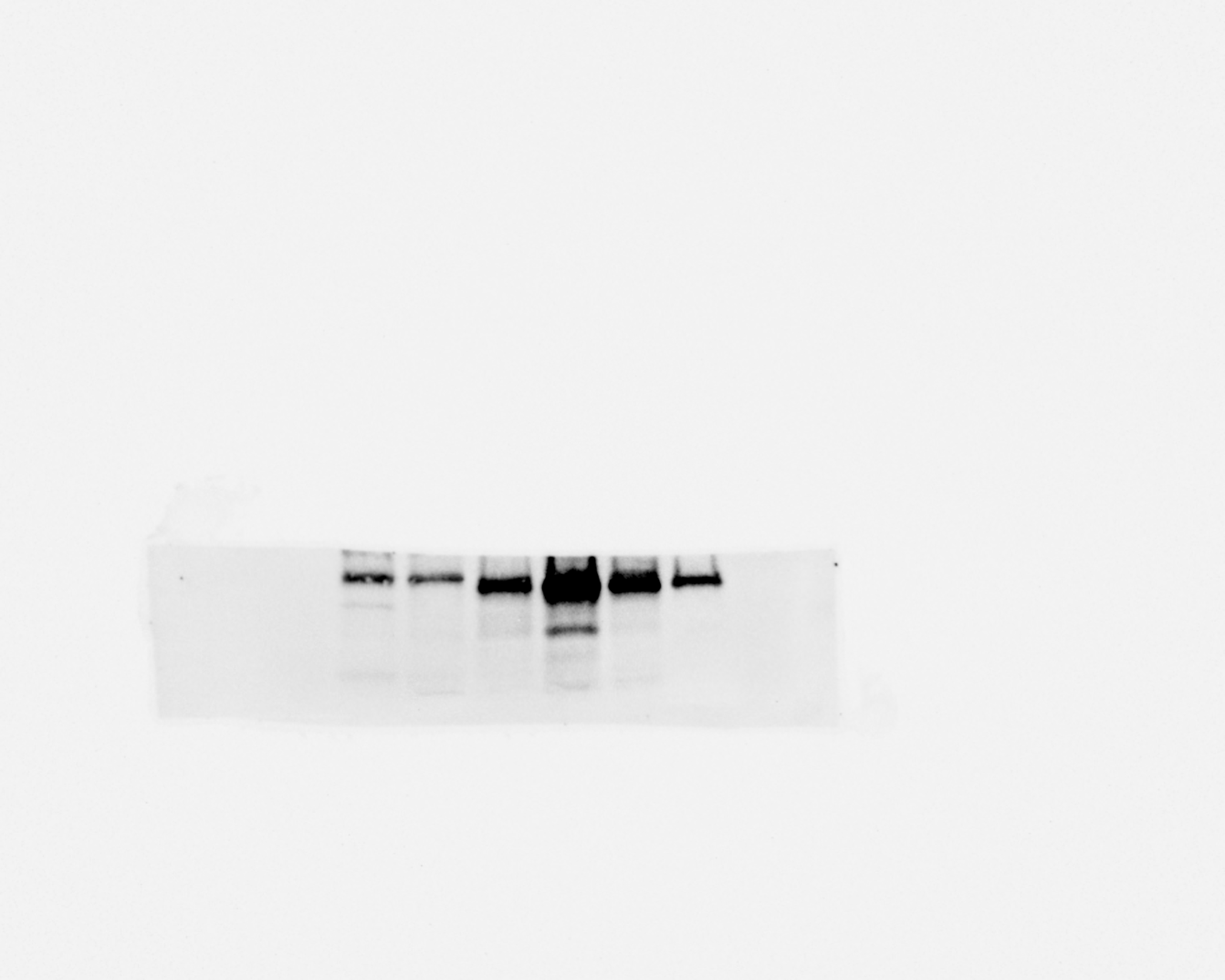


**sFigure 6** Unprocessed anti-HOXB13 blot. The samples are (left to right) H660, DU145, C42B, LNCaP, 22RV1, PC3.


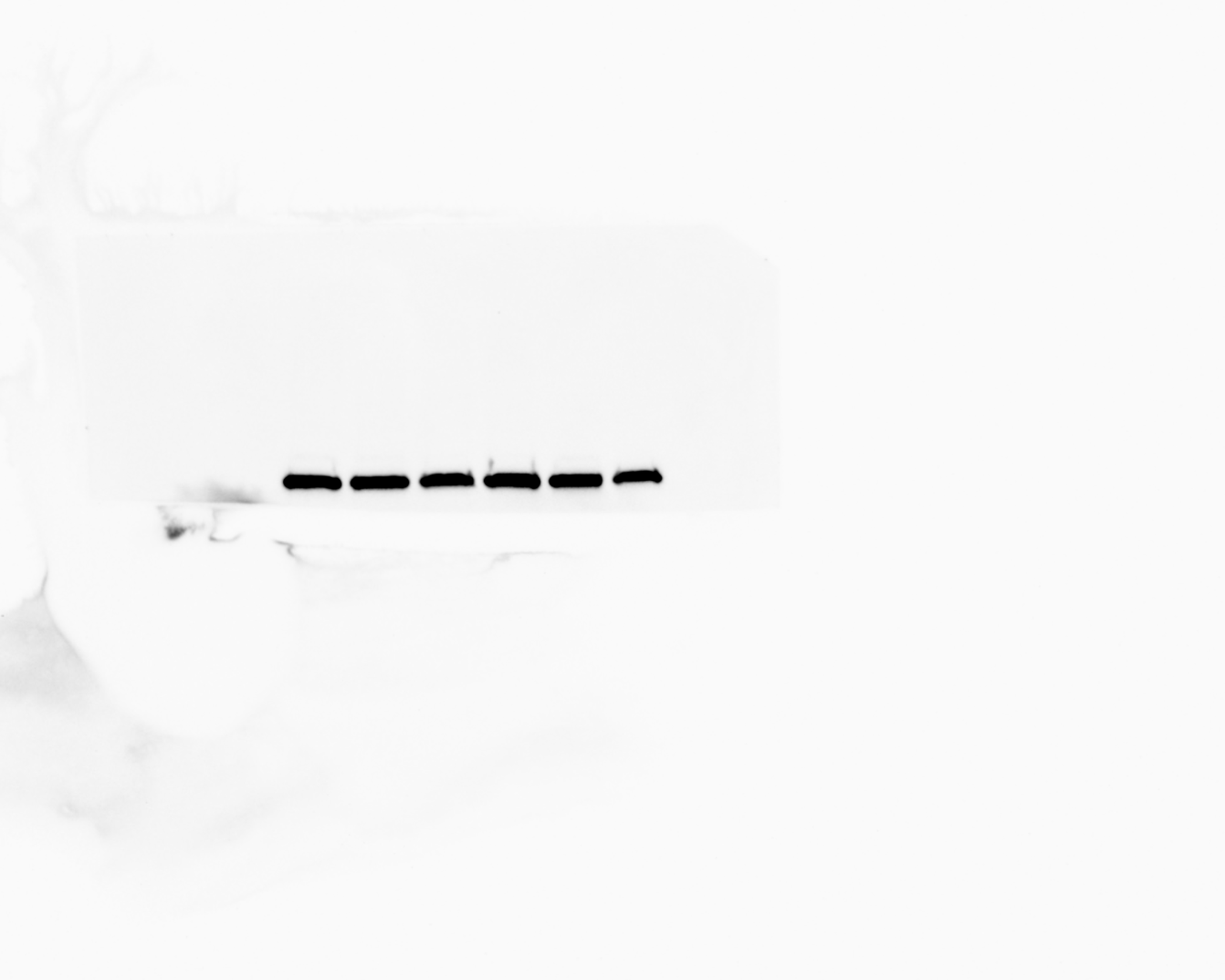


**sFigure 7** Unprocessed anti-b-Tublin blot. The samples are (left to right) H660, DU145, C42B, LNCaP, 22RV1, PC3.
